# Total-body CT and MR features of postmortem change in in-hospital deaths

**DOI:** 10.1371/journal.pone.0185115

**Published:** 2017-09-27

**Authors:** Ivo M. Wagensveld, Britt M. Blokker, Piotr A. Wielopolski, Nomdo S. Renken, Gabriel P. Krestin, Myriam G. Hunink, J. Wolter Oosterhuis, Annick C. Weustink

**Affiliations:** 1 Department of Radiology and Nuclear Medicine, Erasmus University Medical Centre, Rotterdam, Zuid-Holland, The Netherlands; 2 Department of Pathology, Erasmus University Medical Centre, Rotterdam, Zuid-Holland, The Netherlands; 3 Department of Radiology, Reinier de Graaf Gasthuis, Delft, Zuid-Holland, The Netherlands; 4 Centre for Health Decision Science, Harvard T.H. Chan School of Public Health, Harvard University, Boston, Massachusetts, United States of America; University of British Columbia, CANADA

## Abstract

**Objectives:**

To evaluate the frequency of total-body CT and MR features of postmortem change in in-hospital deaths.

**Materials and methods:**

In this prospective blinded cross-sectional study, in-hospital deceased adult patients underwent total-body postmortem CT and MR followed by image-guided biopsies. The presence of PMCT and PMMR features related to postmortem change was scored retrospectively and correlated with postmortem time interval, post-resuscitation status and intensive care unit (ICU) admittance.

**Results:**

Intravascular air, pleural effusion, periportal edema, and distended intestines occurred more frequently in patients who were resuscitated compared to those who were not. Postmortem clotting was seen less often in resuscitated patients (p = 0.002). Distended intestines and loss of grey-white matter differentiation in the brain showed a significant correlation with postmortem time interval (p = 0.001, p<0.001). Hyperdense cerebral vessels, intravenous clotting, subcutaneous edema, fluid in the abdomen and internal livores of the liver were seen more in ICU patients. Longer postmortem time interval led to a significant increase in decomposition related changes (p = 0.026).

**Conclusions:**

There is a wide variety of imaging features of postmortem change in in-hospital deaths. These imaging features vary among clinical conditions, increase with longer postmortem time interval and must be distinguished from pathologic changes.

## Introduction

Hospital autopsy rates today are as low as 0–5%, having decreased from a rate of 30% or higher in the 1990s. [1–3] This low rate is alarming, since one in five autopsies show major discrepancies between antemortem and postmortem diagnoses despite improved diagnostic testing. [4] A possible cause for this decline may be the invasiveness of the conventional autopsy procedure. [5] To provide a less invasive alternative to conventional autopsy, imaging based autopsy methods were developed, primarily in forensic medicine. These modern autopsies include total-body postmortem CT (PMCT) and MR (PMMR), sometimes combined with CT angiography (PMCTA) and image-guided biopsies. [6–8]

More recently the imaging autopsy is steadily emerging in clinical radiology and there is a growing number of diagnostic studies analyzing the performance of the noninvasive (imaging only) and minimally invasive autopsy (imaging with angiography and / or biopsies). [9–11] Combined PMCT, PMCTA and image-guided biopsies appear most sensitive in diagnosing cause of death, however more clinical studies are needed to accurately determine the diagnostic value of the imaging autopsy. [10, 11] In forensic centers access to MR scanners is often limited, so PMCT is most commonly performed. In hospitals, MRI is more widely available, and its high performance to visualize organ parenchyma and soft tissues make PMMR a valuable addition to PMCT.

Postmortem imaging is not the same as imaging the living. Directly after death various chemical and physical processes affect the body in ways that can change PMCT and PMMR features of organs and soft-tissues. These processes can generally be divided into gravity dependent changes (including sedimentation of blood and livor mortis; also known as lividity or hypostasis), decomposition (including putrefaction), rigor mortis (muscle stiffness) and algor mortis (cooling of the body).

Livor mortis is caused by blood settling in the dependent parts of the body due to gravity. Livores can be observed both internally, on imaging and autopsy, and externally upon visual inspection. External livores manifest as dark bluish (or livid) areas of the skin within several hours after death. Internal livores are noted as increased attenuation or signal changes of the dependent areas of organs. The combination of postmortem leakage of cell membranes and subsequent increased osmolality of the interstitial fluid, together with the effect of gravity leads to accumulation of fluids in dependent areas, such as the subcutaneous fat, thoracic cavity and abdominal cavity. [12–14]

Decomposition consists of many processes that cause organic material to break down into simpler forms of matter. It includes putrefaction, autolysis and insect and animal predation. Putrefaction leads to gas formation, it is found intravascular in an early decomposition stage and in more advanced stages also in soft tissues and organ parenchyma.

Rigor mortis leads to muscle contraction after death that results in muscle stiffness. Rigor mortis is caused by cessation of synthesis of adenosine triphosphate (ATP). ATP is consumed in muscle fibers to separate actin and myosin filaments. Directly after death ATP is still present in the muscle, but it is consumed in the first hours after death. When the ATP reserves are depleted, actin and myosin filaments cannot separate anymore. This state lasts until decomposition leads to the breakdown of actin and myosin filaments. The speed of this process depends on temperature: both the time until rigor mortis starts and reaches its maximum and the time until rigor mortis recedes are longer in colder bodies. [15–18]

Algor mortis can affect tissue contrast on PMMR images. There is a wide variability of T1 values due to higher sensitivity of T1 to temperature differences. [19] T2 values are less temperature dependent.

Radiologists need in-depth understanding of these processes for correct acquisition and interpretation of PMCT and PMMR scans. The aim of this study is to evaluate the frequency of total-body CT and MR features of postmortem change in in-hospital deaths.

## Materials and methods

### Study protocol

This study was undertaken as part of the *Minimally Invasive Autopsy* (MIA) study. This is a prospective single center cross-sectional study in a tertiary referral hospital comparing diagnostic performance of conventional autopsy and MIA. Approval of the Erasmus MC Institutional Review Board and Ethics Committee was obtained; the study was filed with the Netherlands National Trial Register. Patients aged 18 years and older who died in the Erasmus University Medical Center were eligible for inclusion, if written informed consent was obtained from next-of-kin for MIA and CA of at least the torso.

Exclusion criteria were (suspected) unnatural COD, body size exceeding diameter of 16 inches in supine position (limitation for PMMR), known or suspected “high-risk” infected bodies (tuberculosis, hepatitis B and C, human immunodeficiency virus, methicillin-resistant Staphylococcus aureus, multi-drug resistant Acinetobacter), and open abdominal wounds that could not be completely closed or taped to prevent leakage of body fluids.

All cases underwent total-body PMCT and PMMR followed by biopsies under CT (torso) or stereotactic guidance (brain) according to standardized protocols (Tables 1 and 2). Total scan time was approximately 60 minutes for PMMR and 10 minutes for PMCT. First PMMR was performed on a 1.5T scanner (Discovery MR450, GE Healthcare, Milwaukee, Wisconsin USA) and consisted of scans from the head to the pelvis (legs were omitted on because of MR scanner availability). Directly after PMMR, PMCT was performed on a dual-source CT scanner (SOMATOM Definition Flash, Siemens Healthcare Forchheim, Germany) and consisted of scans from head to feet. Standardized CT-guided biopsies (12 Gauge) were taken from heart, lungs, liver, kidneys, spleen, and additional biopsies were taken from radiologically suspected pathology. All biopsies were stained with hematoxylin and eosin (H&E) and when requested by the pathologist additional stains were performed.

**Table 1:** Postmortem imaging protocol.

A. Postmortem magnetic resonance protocol										
*Scan area*	***Coil***	***Sequence***	***TR/TE/TI*** ***(ms)***	***Slice width (mm)***	***FOV*** ***(cm)***	***Matrix***	***Number of slices***	***Coverage per section (cm)***	***Number of sections***	***Scan time per section (s)***
Head-Pelvis	Body	FLAIR FSE T1w	2320/9.5/963	4.0	48	384x320	50	20.0	5–8	174
Head-Pelvis	Body	STIR FSE T2w	12000/41/120	4.0	48	288x224	50	20.0	5–8	168
Thorax	8-channel	3D fs FSPGR T1w	3.3/1.2/14	1.6	40	256x256	212	33.9	1	153
Thorax	8-channel	2D STIR FSE T2w	11200/ 94/120	2.0	40	256x256	170	34.0	1	359
B. Postmortem computed tomography protocol										
*Scan area*	***Rotation time*** ***(s)***	***Tube voltage*** ***(kV)***	***Tube current*** ***(eff*. *mAs)***	***Slice collimation*** ***(mm)***	***Pitch***	***Scan time (s)***	***Reconstruction***			
Head-Neck	1.0	100	750	2 x 64 x 0.6	0.35	21	FBP			
Thorax-Abdomen	1.0	120	600	2 x 64 x 0.6	0.6	32	FBP			
Extremities	1.0	120	600	2 x 64 x 0.6	0.6	57	FBP			

**Table 2:** Image-guided biopsy protocol.

Organ	Targets aimed at
**Brain**	Normal parenchyma and suspected pathology
**Lungs**	Both lungs, normal parenchyma and suspected pathology
**Heart**	Left ventricle: lateral wall, apex, normal myocardium and suspected pathology. Right ventricle: on indication
**Kidneys**	Both kidneys, normal parenchyma and suspected pathology
**Spleen**	Sub-capsular parenchyma and suspected pathology
**Liver**	Normal parenchyma and suspected pathology
**Other**	Region of interest

### Scoring

We composed a scoring list of PMCT and PMMR features of postmortem change (Tables 3–5). The features that were included were based on our radiological expertise [9, 10] and were supplemented with features from published postmortem imaging studies. [5, 12, 20–51]

**Table 3:** Scoring list of PMCT and PMMR features of postmortem changes in the brain.

Postmortem imaging feature	Category	Postmortem process
1. **Hyperdense superior sagittal sinus**	Gravity dependent changes	Blood sedimentation
2. **Hyperdense veins**	Gravity dependent changes	Blood sedimentation
3. **Hyperdense arteries**	Gravity dependent changes	Blood sedimentation
4. **Thickened / irregular falx**	Gravity dependent changes	Blood sedimentation
5. **Liquid in paranasal sinus**	Decomposition	Cell wall leakage
6. **Intracranial air**	Decomposition	Putrefaction
7. **High T1 signal basal ganglia**	Algor mortis	Temperature change
8. **Low T2 signal basal ganglia**	Algor mortis	Temperature change
9. **Diffusion restriction**	Algor mortis	Temperature change
10. **Insufficient suppression of liquor on Flair**	Algor mortis	Temperature change
11. **Loss of grey-white matter differentiation**	Miscellaneous	Cerebral hypoxia
12. **Sulcal effacement**	Miscellaneous	Cerebral hypoxia
13. **Compression cisterns**	Miscellaneous	Cerebral hypoxia
14. **Cerebellar tonsillar herniation**	Miscellaneous	Cerebral hypoxia

**Table 4:** Scoring list of PMCT and PMMR features of postmortem changes in the thorax.

Postmortem imaging feature	Category	Postmortem process
1. **Hyperdense aortic wall**	Gravity dependent	Blood sedimentation
2. **Sedimentation of blood aorta**	Gravity dependent	Blood sedimentation
3. **Sedimentation of blood large vessels**	Gravity dependent	Blood sedimentation
4. **Livores heart**	Gravity dependent	Livor mortis
5. **Sedimentation of blood heart**	Gravity dependent	Blood sedimentation
6. **Livores lung**	Gravity dependent	Livor mortis
7. **Groundglass opacification**	Gravity dependent	Livor mortis
8. **Increased density dependent areas skin and subcutis**	Gravity dependent	Livor mortis
9. **Edema dependent areas subcutis**	Decomposition / Gravity dependent	Cell wall leakage / Livor mortis
10. **Intravascular air**	Decomposition	Putrefaction
11. **Gas formation heart**	Decomposition	Putrefaction
12. **Gas formation myocardium**	Decomposition	Putrefaction
13. **Susceptibility artifacts heart (gas)**	Decomposition	Putrefaction
14. **Pericardial effusion**	Decomposition	Cell wall leakage
15. **Pleural effusion**	Decomposition	Cell wall leakage
16. **Gas formation lung parenchyma**	Decomposition	Putrefaction
17. **T2 signal decay from subepicardial to subendocardial**	Rigor mortis	Rigor mortis
18. **Dilated vena cava inferior**	Miscellaneous	Loss of blood pressure
19. **Postmortem clotting large vessels**	Miscellaneous	Clotting
20. **Collapse large vessels**	Miscellaneous	Loss of blood pressure
21. **Dilated heart**	Miscellaneous	Loss of blood pressure
22. **Dilated right atrium**	Miscellaneous	Loss of blood pressure
23. **Postmortem clotting heart**	Miscellaneous	Clotting
24. **Collapse of aorta**	Miscellaneous	Loss of blood pressure
25. **Postmortem clotting aorta**	Miscellaneous	Clotting
26. **Dilated vena cava superior**	Miscellaneous	Loss of blood pressure
27. **Gas formation subcutaneous areas**	Decomposition	Putrefaction
28. **Liquid trachea / bronchi**	Decomposition	Cell wall leakage

**Table 5:** Scoring list of PMCT and PMMR features of postmortem changes in the abdomen.

Postmortem feature	Category	Postmortem process
1. **Intestinal sedimentation**	Gravity dependent	Sedimentation
2. **Livores liver**	Gravity dependent	Livor mortis
3. **Sedimentation gall bladder**	Gravity dependent	Sedimentation
4. **Livores spleeny**	Gravity dependent	Livor mortis
5. **Livores kidneys**	Gravity dependent	Livor mortis
6. **Free air**	Decomposition	Putrefaction
7. **Fluid in the abdomen**	Decomposition	Cell wall leakage
8. **Gas in the intestinal wall**	Decomposition	Putrefaction
9. **Distended intestines**	Decomposition	Putrefaction
10. **Gas liver parenchyma**	Decomposition	Putrefaction
11. **Air liver vessels**	Decomposition	Putrefaction
12. **Gas bile ducts**	Decomposition	Putrefaction
13. **Periportal edema**	Decomposition	Cell wall leakage
14. **14. Gas spleen parenchyma**	Decomposition	Putrefaction
15. **Gas kidney parenchyma**	Decomposition	Putrefaction
16. **Intravascular air**	Decomposition	Putrefaction
17. **Collapse aorta**	Miscellaneous	Loss of blood pressure
18. **Collapse vena cava**	Miscellaneous	Loss of blood pressure
19. **Dilated vena cava**	Miscellaneous	Loss of blood pressure

All cases were retrospectively and independently scored by a radiologist (ACW; board-certified with 10 years of clinical expertise in postmortem imaging) and a researcher (IMW with 3 years of expertise). When available, clinical information and antemortem scans were reviewed. Specific clinical conditions were scored; including intensive care unit (ICU) admittance and post-resuscitation status (PRS).

PMCT and PMMR features were–if possible–categorized to a specific chemical and/or physical process; 1. gravity dependent changes; 2. decomposition; 3. rigor mortis; 4. algor mortis. Features that could not be classified to any of these four processes were labeled to a miscellaneous category.

### Statistical analyses

We recorded percentage of male/female cases, mean age at death, and mean postmortem time interval (PTI) including standard deviations. PTI was defined as the time from death to the start of MR scanning. For each case, we calculated the frequency of PMCT and PMMR features. Fisher’s exact test was used for the association between specific clinical conditions (ICU and PRS) and frequencies of PMCT and PMMR features. Linear discriminant analysis was used to evaluate the correlation of PTI and PMCT and PMMR features. ANOVA was used to test the correlation of PTI and a combination score for all decomposition and all gravity dependent changes. The inter-observer agreement was calculated using kappa statistics (agreement <0.2: poor, 0.2–0.4: fair, 0.4–0.6: moderate, 0.6–0.8: good, and 0.8–1.0: very good). Furthermore we calculated inter-observer agreement for the group of pathological mimics (those postmortem changes that were most likely to be confused with real pathologic changes) and a group of postmortem changes that does not correspond to a pathologic process with similar radiological features.

## Results

We scanned 100 cases from January 2012 to December 2014. The mean age was 62.7 (±13.0), 62% were male (Table 6). Inter-observer agreement was very good, with a kappa of 0.84 for PMCT and 0.83 for PMMR. The kappa score for the group with pathological mimics was 0.79 for PMCT and 0.76 for PMMR, the kappa score for the non-pathologic mimics was 0.89 for PMCT and 0.88 for PMMR.

**Table 6:** Patient demographics.

**Patient demographics (n = 100)**				
Men/women (n)	62/38			
Mean age (SD, min-max)	62.7 (±13.0, 25–92)			
Mean PTI (SD, min-max)	22.6 (±15.4, 3.1–71.5)			
PRS (n)	43			
Mean image acquisition time	MRI: 59 minutes, CT: 3–4 minutes			
**Hospital ward**	**n**			
ICU	38			
ER	15			
Internal medicine / gastroenterology	11			
Oncology	8			
Neurology	6			
Thoracic surgery	5			
Hematology	5			
Pulmonology	5			
General surgery	4			
Gynecology/urology	3			
**Antemortem imaging**	Not available (n)	CT (n)	MR (n)	CT and MR (n)
Brain	65	26	5	4
Thorax	30	70	0	0
Abdomen	31	67	0	2

SD = standard deviation; PTI = postmortem time interval (hours); PRS = post-resuscitation status; ICU = intensive care unit; ER = emergency room; CT = computed tomography, MR = magnetic resonance

### Total-body CT and MR features of postmortem change–general overview

PMCT and PMMR features of different organs and observed frequencies are presented in Tables 7–9.

**Table 7:** Frequencies of postmortem PMCT and PMMR features of the brain.

PMCT and PMMR features of brain (n = 100)		
	**PMCT**	**PMMR**
**Loss of grey-white matter differentiation**	85%	85%
**Hyperdensity superior sagittal sinus**	96%	NA
**Hyperdensity veins**	54%	NA
**Sulcal effacement**	44%	41%
**Hyperdensity Willis' circle and cerebral arteries**	35%	NA
**Liquid paranasal sinuses**	32%	32%
**High T1 signal basal ganglia and thalamus**	NA	32%
**Thickened / irregular aspect falx**	20%	NA
**Intracranial air cerebral vasculature**	8%	1%

PMCT = postmortem CT; PMMR = postmortem MR; NA = not assessable

**Table 8:** Frequencies of postmortem PMCT and PMMR features of thorax.

PMCT and PMMR features of thorax (n = 100)		
***Heart and large vessels***	**PMCT**	**PMMR**
**Air heart**	44%	22%
**Sedimentation of blood heart**	62%	84%
**Dilatation right atrium and ventricle**	25%	25%
**Pericardial effusion**	26%	27%
**T2 signal decay epi- to endocardial**	NA	12%
**Postmortem clotting heart**	4%	8%
**Air pericardial space**	4%	2%
**Air coronaries**	18%	12%
**Hyperdense aortic wall**	90%	NA
**Sedimentation in blood vessels**	71%	88%
**Postmortem clotting vessels**	25%	38%
**Intravascular air**	31%	8%
**Collapse of thoracic aorta**	30%	29%
***Lungs***	**PMCT**	**PMMR**
**Internal livores**	86%	85%
**Pleural effusion**	31%	38%
**Liquid trachea / main bronchi**	78%	78%

PMCT = postmortem CT; PMMR = postmortem MR; NA = not assessable

**Table 9:** Frequencies of postmortem PMCT and PMMR features of the abdomen.

PMCT and PMMR features of abdomen (n = 100)			
***Liver*, *gallbladder*, *spleen and kidney***	**PMCT**		**PMMR**
**Gas liver vasculature**	37%		26%
**Internal livores liver**	NA		74%
**Sedimentation gallbladder**	8%		14%
**Periportal edema**	11%		27%
**Internal livores spleen**	NA		31%
**Gas spleen parenchyma or vessels**	5%		1%
**Internal livores kidneys**	NA		6%
**Gas kidney parenchyma or vessels**	9%		1%
***Stomach*, *intestines*, *abdominal cavity***	**PMCT**		**PMMR**
**Intestinal sedimentation**	6%		15%
**Gas in the intestinal wall**	8%		1%
**Distended intestines**	14%		14%
**Free air**	7%		2%
**Fluid in the abdomen**	20%		35%
***Abdominal vessels***	**PMCT**		**PMMR**
**Intravascular air**	21%		5%
**Collapse abdominal aorta**	67%		67%
**Collapse abdominal vena cava**	53%		53%
**Dilated abdominal vena cava**	2%		2%
**Air vertebral venous plexus**	11%		2%

PMCT = postmortem CT; PMMR = postmortem MR; NA = not assessable

#### Brain

**Fig 1:**
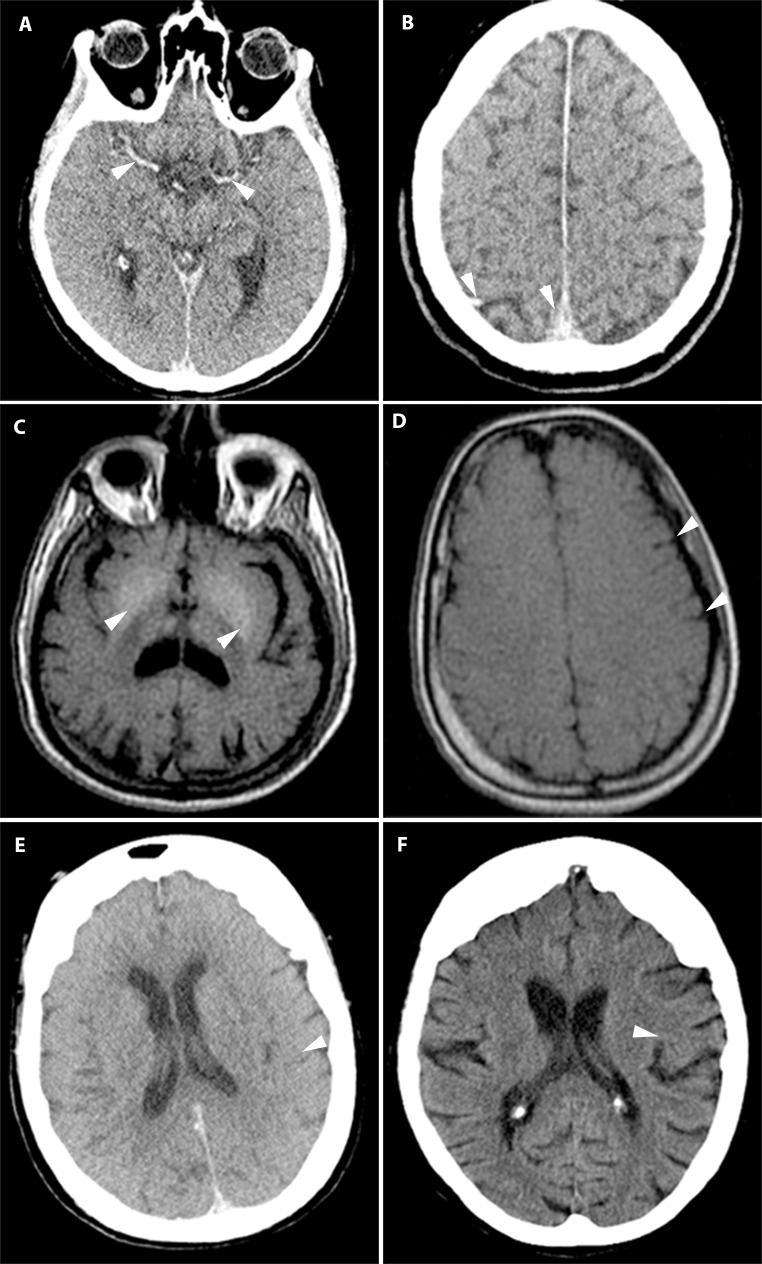
Postmortem imaging features of the brain. (A) PMCT: Symmetrical hyperdense cerebral arteries (arrowheads). (B) PMCT: Hyperdensity in the dependent cerebral veins and sagittal sinus (arrowheads). (C) T1w PMMR: High signal of the basal nuclei of the brain (arrowheads). (D) T1w PMMR: Sulcal effacement (arrowheads). (E/F) PMCT (E) and antemortem CT (F) of the same patient. The antemortem CT scan shows normal grey-white matter differentiation (arrowhead). PMCT shows complete loss of grey-white matter differentiation (arrowhead).

Sedimentation led to increased attenuation of the posterior sagittal sinus (96%), cerebral veins (54%) and cerebral arteries (35%) in a symmetric distribution (Fig 1A and 1B). Putrefactive gas in the brain was seen in only a few cases (8%). Liquefaction of the brain was not observed. PMMR showed high T1 signal of the basal ganglia in one third of cases (Fig 1C). Effacement of sulci (Fig 1D) and loss of grey-white matter differentiation was seen in the majority of cases (85%) (Fig 1E and 1F).

#### Heart and large vessels

**Fig 2:**
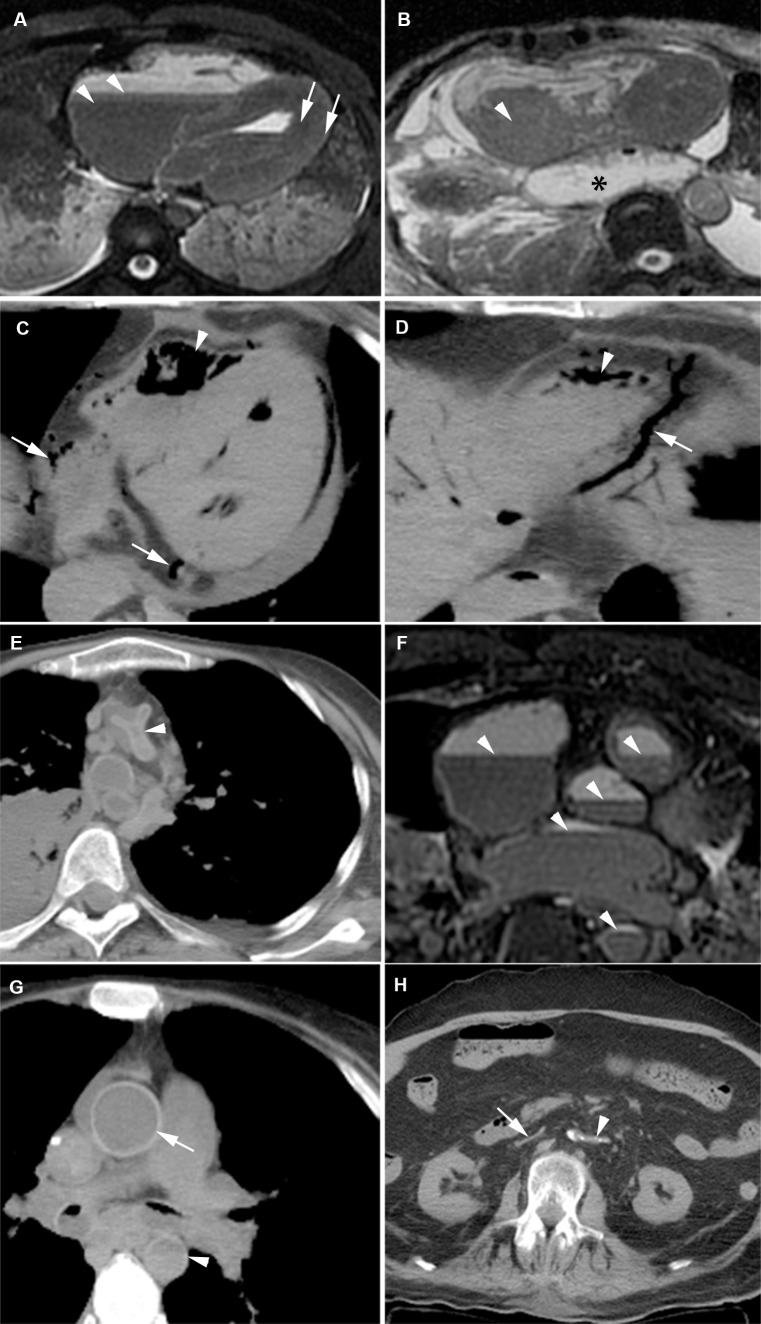
Postmortem imaging features of the heart and large vessels. (A) T2w PMMR: Sedimentation of blood in the heart chambers (arrowhead). T2 signal decay from subepicardial to subendocardial myocardium (arrows). (B) T2w PMMR: Postmortem clotting in the right atrium (arrowhead). Additional finding: a mediastinal herniation of the stomach (asterisk). (C/D) PMCT: Extensive air in the right and left ventricle (arrowheads) and coronary veins (arrows). (E) PMCT: Collapsed ascending aorta (arrowhead). (F) T2w PMMR: Sedimentation of the blood (arrowheads), the plasma layer becomes hyperintense and the dependent layer becomes hypointense. (G) PMCT: Relatively hyperdense aortic wall (arrow) as a result of sedimentation. This is best seen in the ascending aorta. The descending aorta shows a sedimentation level with a hyperdense aspect of the anterior vessel wall (arrowhead). (H) PMCT: Complete collapse of the abdominal aorta (arrowhead) and vena cava inferior (arrow).

The right atrium and ventricle were dilated in 25% (Fig 2A). The thoracic aorta showed clotting in 38% of cases and was detected best on PMMR (Fig 2B). Air in the heart chambers was seen in 44% (Fig 2C and 2D). No air was observed within the myocardium. T2 signal decline in the myocardium from the epicardial to endocardial regions was seen in 12% (Fig 2A).We observed a collapse of the thoracic aorta in 30% (Fig 2E). Sedimentation of blood was often present in the heart (84%) (Fig 2A) and large thoracic vessels (Fig 2F). The thoracic aortic wall showed increased attenuation in a majority of cases (90%) (Fig 2G). The abdominal aorta was collapsed in 67% and the abdominal vena cava in 53% (Fig 2H). Air in the vertebral venous plexus was seen less frequently (11%), and usually in cases with extensive intravascular air.

#### Lungs

**Fig 3:**
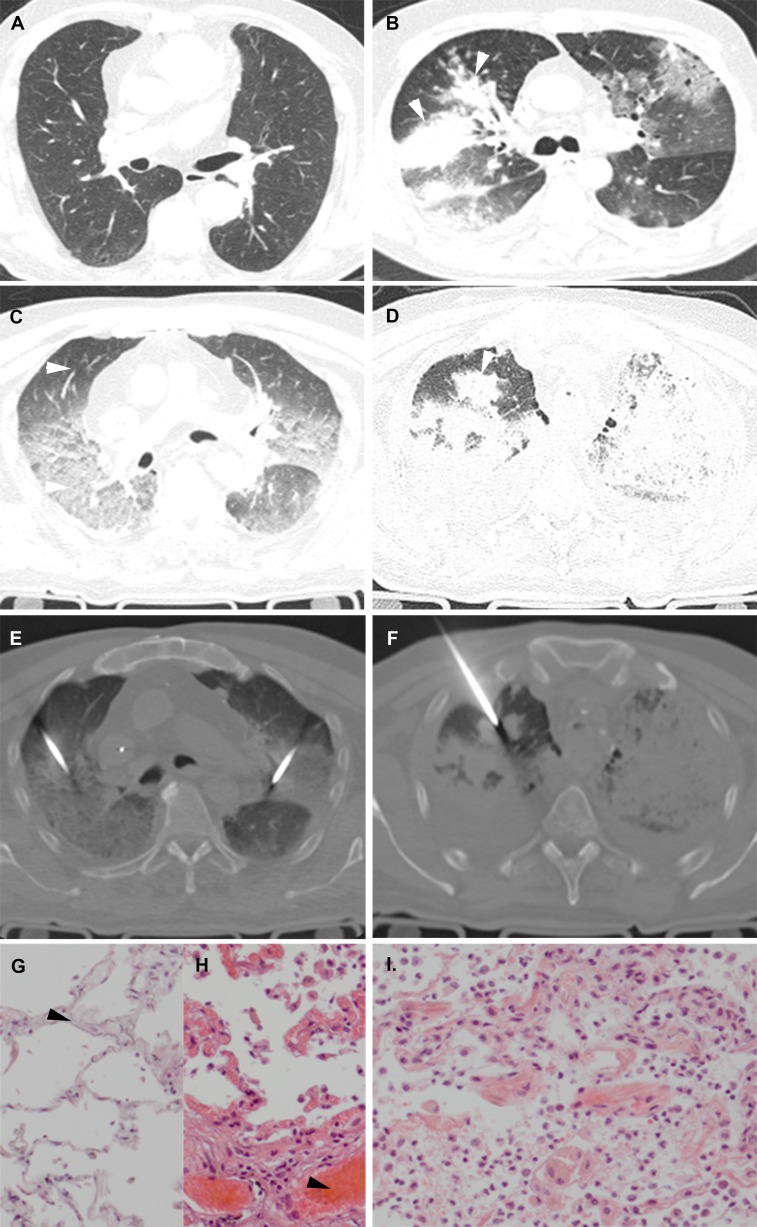
Internal livores of the lungs versus pneumonia. (A/C) antemortem CT (A) and PMCT (C). Normal (A) and internal livores (C) (arrowheads). (B/D) antemortem CT (B) and PMCT (D). Patient with a pneumonia (arrowheads). (E) CT-guided lung biopsies in the same patient as A/C. (F) CT-guided lung biopsy in the same patient as B/D. (G) HE, x100 original magnification. Lung parenchyma non-dependent: capillaries in alveolar walls practically devoid of blood (arrowhead). (H) HE, x100 original magnification. Same patient as G, lung parenchyma dependent, capillaries in alveolar walls congested with blood (arrowhead). (I) HE, x100 original magnification. Same patient as B/D/F, lung parenchyma, resolving pneumonia with thickened alveolar walls with mainly lymphocytic infiltrates, and hyaline membranes and extravasations of erythrocytes in the alveolar spaces.

Livor mortis affected the lungs frequently (86%); it appeared as areas with increased density or high T2 signal in the dependent areas of the lungs. In these parts of the lung it is challenging to distinguish livores from pneumonia (Fig 3A–3I) or other interstitial diseases. Liquid in the trachea and bronchi was very common (78%). Pleural effusion was seen in only 38% of cases.

#### Liver, spleen, kidneys, gallbladder, pancreas, adrenals

**Fig 4:**
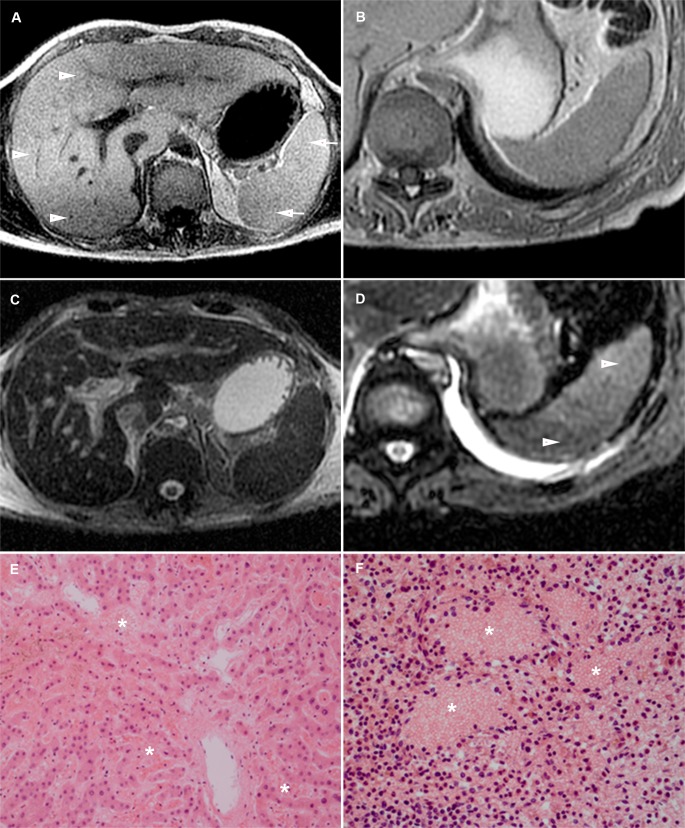
Internal livores of the liver and spleen. (A/C) T1w (A) and T2w fs (C) PMMR. Internal livores. In the liver 3 distinct layers (arrowheads) of different T1 signal can be seen and in the spleen 2 layers (arrows). A low T1 signal layer on top, relatively intermediate-to high signal layer in the middle and a low T1 signal layer in the dependent part of the liver. On T2w fs internal livores are not clearly seen. (B/D) T1w (B) and T2w fs (D) PMMR. The spleen shows 2 layers of different T2 signal (arrowheads). T1w show no clear livores in this patient. (E) HE, x100 original magnification. Centrilobular area of the liver with wide sinuses extended by blood (asterisks). (F) HE, x200 original magnification. Congested spleen with lakes of blood (asterisks).

**Fig 5:**
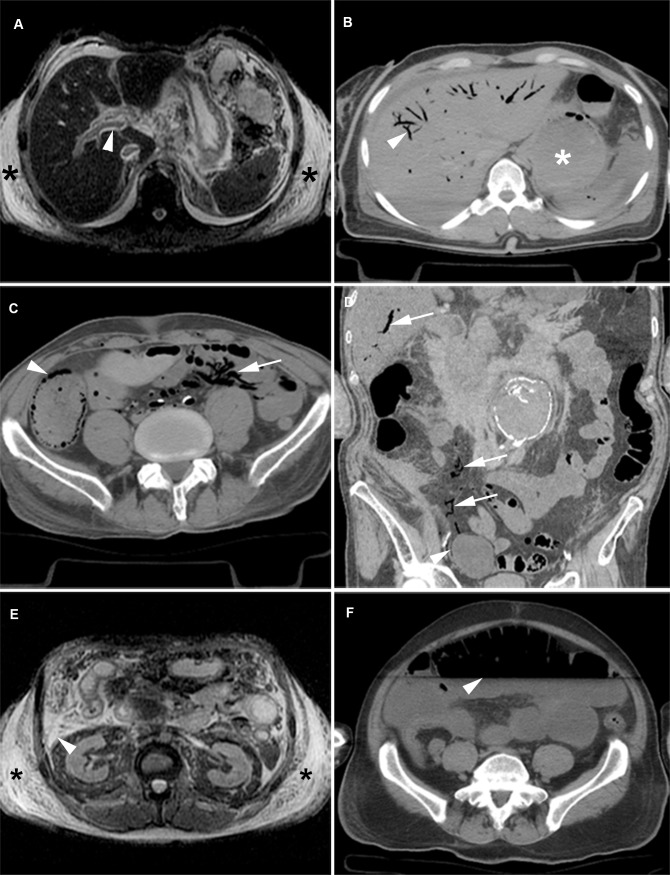
Postmortem imaging features of the abdomen. (A) T2w PMMR: Periportal edema (arrowhead) and subcutaneous edema (asterisks). (B) PMCT: Putrefactive gas in the liver vessels (arrowhead) and distended stomach (asterisk). (C/ D) PMCT: Gas in the intestinal wall (arrowhead) and air in the mesenteric and portal veins (arrows). (E) T2w PMMR: Subcutaneous edema (asterisks) and fluid in the abdomen (arrowhead). (F) PMCT: Distended intestines with a sharp fluid-air level (arrowhead).

Internal livores of the spleen and kidney were noted by two layers of different T1 and T2 signal reflecting blood settling in the parenchyma. In the liver, three layers can be seen: an upper layer with small amounts of putrefactive gas, a middle layer with intermediate signal and a lower layer that together with the middle layer reflect settling of blood (Fig 4A–4F). Gravity can cause sedimentation of the gallbladder content and this is best seen on PMMR as vertical signal gradients. Livor mortis in organ parenchyma (spleen 31%, kidneys 6% and liver 74%) was also best depicted on PMMR and presented as different layers of T1 and T2 signal. In general, livores of the organ parenchyma were not clearly detectable on PMCT. Periportal edema was found on PMMR in 27% (Fig 5A). Putrefaction gas in the liver vasculature was seen on PMCT in 37% (Fig 5B). The imaging features of the pancreas and adrenal glands were not notably affected by postmortem change.

#### Stomach, intestines, abdominal cavity

Sedimentation in the stomach and intestines was seen in only a few cases (15%). Fluid in the abdomen was present in 35%. Bowel distension (14%), gas in the intestinal wall (8%) and free abdominal air (7%) were less common features (Fig 5C–5F).

#### Soft tissues

On PMCT superficial internal livor mortis was manifested as increased densities of the dependent subcutaneous areas (37%). [12] Putrefactive gas in subcutaneous tissue was not observed.

### Total-body CT and MR features of postmortem change–in relation to clinical conditions and postmortem time interval

#### Intensive care unit admittance

In our cohort 38/100 patients died in the ICU. Livores of the liver was seen significantly more often in ICU patients than in non-ICU patients (92% vs. 62%, p = 0.001) (Table 10). High T1 signal of the basal ganglia was significantly less frequently observed in ICU patients (44% vs. 13%, p = 0.001).

**Table 10:** Postmortem CT and MR features in relation to clinical conditions.

Clinical condition	Modality*	Yes	No	Total	P-value
***Intensive care unit admittance***		**N = 38**	**N = 62**	**N = 100**	
**Hyperdense cerebral arteries**	PMCT	17 (45%)	18 (29%)	35 (35%)	0.133
**High T1 signal basal ganglia**	PMMR	5 (13%)	27 (44%)	32 (32%)	0.002
**Postmortem clotting**	PMMR	21 (55%)	23 (37%)	44 (44%)	0.098
**Subcutaneous edema**	PMMR	17 (45%)	20 (32%)	37 (37%)	0.286
**Fluid in the abdomen**	PMMR	17 (45%)	18 (29%)	35 (35%)	0.133
**Livores liver**	PMMR	35 (92%)	39 (63%)	74 (74%)	0.001
**Livores spleen**	PMMR	10 (26%)	21 (34%)	31 (31%)	0.507
***PRS***		**N = 43**	**N = 57**	**N = 100**	
**Pleural effusion**	PMMR	25 (58%)	13 (23%)	38 (38%)	<0.001
**Periportal edema**	PMMR	19 (44%)	8 (14%)	27 (27%)	0.001
**Distended intestines**	PMCT/PMMR	9 (21%)	5 (9%)	14 (14%)	0.144
**Postmortem clotting**	PMMR	11 (26%)	33 (58%)	44 (44%)	0.002
**Dilated right atrium / ventricle**	PMCT/PMMR	15 (35%)	10 (18%)	25 (25%)	0.063
**Intravascular air**	PMCT	31 (72%)	27 (47%)	58 (58%)	0.015

*Specifies the modality that has the highest detection of the postmortem changes

PMCT = postmortem CT; PMMR = postmortem MR; PRS = post-resuscitation status

#### Post-resuscitation status

Forty-three patients underwent unsuccessful resuscitation just prior to death. Pleural effusion (p<0.001) and periportal edema (p = 0.001) were seen significantly more often in patients that had undergone resuscitation (Table 10). Postmortem clotting occurred significantly less frequently in patients that had underwent resuscitation (p = 0.002). Intravascular air (both arterial and venous) was visible in 58% of patients and more frequently present in PRS patients than in non-PRS patients (72% vs. 47%, p = 0.013).

#### Postmortem time interval

The mean PTI was 23.0 (±15.6) hours. PTI showed a significant correlation with internal livores of the lungs (p = 0.038), distended intestines (p = 0.001) and loss of grey-white matter differentiation in the brain (p<0.001) (Table 11). PTI showed a significant correlation with postmortem changes related to decomposition (p = 0.026).

**Table 11:** Postmortem CT and MR features in relation to postmortem time interval.

Postmortem time interval	Modality*	<12 hours	12–24 hours	24–48 hours	>48 hours	Total	P-value
		**N = 25**	**N = 37**	**N = 28**	**N = 10**	**N = 100**	
**Intravascular air**	PMCT	14 (56%)	23 (62%)	16 (57%)	5 (50%)	58 (58%)	0.905
**Sedimentation blood**	PMMR	24 (96%)	35 (95%)	28 (100%)	10 (100%)	97 (97%)	0.416
**Loss of grey-white matter differentiation**	PMCT/PMMR	14 (56%)	33 (89%)	28 (100%)	10 (100%)	85 (85%)	<0.001
**Distended intestines**	PMCT/PMMR	0 (0%)	4 (11%)	7 (25%)	3 (30%)	14 (14%)	0.001
**Postmortem clotting**	PMMR	6 (24%)	20 (54%)	12 (43%)	6 (60%)	44 (44%)	0.197
**Livores lungs**	PMCT/PMMR	20 (80%)	30 (81%)	26 (93%)	10 (100%)	86 (86%)	0.038
**Livores liver**	PMMR	17 (68%)	27 (73%)	25 (89%)	5 (50%)	74 (74%)	0.805
**Livores spleen**	PMMR	5 (20%)	11 (30%)	10 (35%)	5 (50%)	31 (31%)	0.062
**Gravity dependent changes**	PMCT/PMMR	50%	47%	51%	56%	50%	0.094
**Decomposition**	PMCT/PMMR	23%	30%	29%	34%	28%	0.026

*Specifies the modality that has the highest detection of the postmortem change

PMCT = postmortem CT; PMMR = postmortem MR

## Discussion

This is the first study evaluating the frequency of PMCT and PMMR features of postmortem change in a large cohort of adult patients. Similar imaging studies on postmortem change in fetuses and neonates have been published. [13, 52]. We observed a wide variety of PMCT and PMMR features of postmortem change. Particularly livor mortis and decomposition have great impact on the imaging features. Algor mortis and rigor mortis lead to only minor changes. Our results indicate that PMCT and PMMR appear to be complementary for correct interpretation of postmortem changes. Some changes are more clearly seen on PMMR such as livores of organ parenchyma or blood clotting, while others such as the presence and distribution of putrefaction air is better noted on PMCT.

Clinical conditions may influence imaging features of postmortem change. Importantly, postmortem changes may mimic or even mask real pathological changes related to the cause of death: e.g. gravity causes sedimentation of blood contents within the first hours after death. On PMCT the upper (plasma) and lower layer (blood cells) shows decreased and increased attenuation respectively. As a result the upper part of the aortic wall shows relatively high attenuation compared to the plasma content and may mimic aortic wall hematoma (Fig 2G).

Bacterial infections can speed decomposition processes and increase gas and fluid formation in the body. Hypovolemia causes the heart cavities and vessel lumen to decrease in size.

Medical treatments can also change imaging features; e.g. intravascular lines and surgical wounds can be accompanied by air in the surrounding soft tissues and bloodstream.

Resuscitation can cause rib fractures, pneumothorax, lung contusions, hemothorax, and intravascular air. We found that the majority of PRS patients showed significantly more intravascular air as opposed to non-PRS patients, suggesting that air was introduced during resuscitation. [12, 27, 53, 54] Intravascular air after resuscitation is caused by pneumatization of dissolved gas in the blood as a result of compression and expansion of vessels and direct mechanical force to the chest allowing air from the lungs to enter the bloodstream. [55, 56] Likewise resuscitation attempts may introduce free abdominal air that should not be confused with free air caused by intestinal perforation. [13] Pleural effusion, periportal edema, and distended intestines were also more frequently observed after resuscitation. Postmortem clotting occurred less often in PRS patients and we hypothesize this is caused by anti-coagulation given during resuscitation attempts. [12, 27, 53, 54]

Lack of oxygenation in the brain was noted by loss of grey- white matter differentiation, edema, swelling of the brain and effacement of sulci. [23, 57, 58] These features involve the entire brain and are symmetrical. [14, 23] Patients with elevated intracranial pressure prior to death may show similar features, and comparison to antemortem scans is recommended. In living patients a dense-artery-sign in the cerebral arteries is often asymmetric and indicative for cerebral ischemia, a postmortem mimic of this sign is usually symmetrical and is non-pathological (Fig 1A). The cessation of cardiac output and fall in blood pressure causes the arterial wall to collapse directly after death. [30, 59] This change may obscure an aortic aneurysm or dissection. Within 2 hours after death blood clots form in the heart and large vessels.

**Fig 6:**
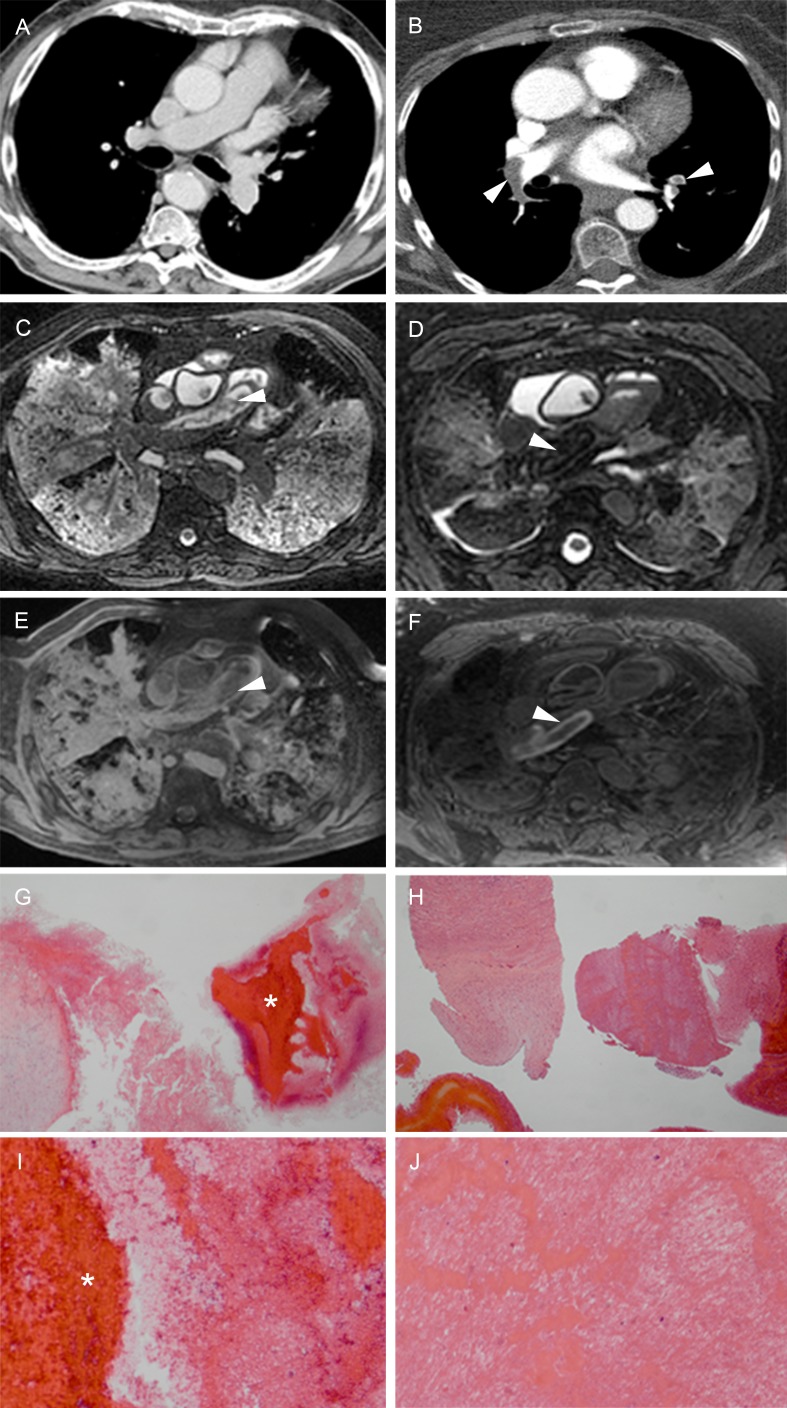
Pulmonary thrombo-embolism versus postmortem blood clot. (A/C/E) Antemortem CT (A) and T2w (C) and T1w (E) PMMR in the same patient. Antemortem CT (A) shows no abnormalities in the pulmonary arteries. PMMR (C/E) shows an irregularly shaped clot in the right pulmonary artery (C: arrowhead), with low T1 signal (E: arrowhead). (B/D/F) Antemortem CTA (B) and T2w (D) and T1w (F) PMMR in a patient with a thrombo-embolus. Antemortem CTA (B) shows a thrombo-embolus in the right and left pulmonary artery (arrowheads). PMMR (D/F) shows a thrombo-embolus in the right pulmonary artery (arrowheads). (G) HE, x16 original magnification, corresponding to image A/C/E. Wall of right pulmonary artery with a postmortem blood clot (asterisk). (I) HE, x50 original magnification, corresponding to image A/C/E. Higher power of blood clot showing blood with loosely arranged depositions of fibrin. (H) HE, x16 original magnification, corresponding to image B/D/F. Wall of pulmonary artery and thrombus. (J) HE, x50 original magnification, corresponding to image B/D/F. Wall of pulmonary artery and thrombus with dense depositions of fibrin and platelets, alternating with degenerated erythrocytes and white blood cells, thus constituting so-called lines of Zahn.

Postmortem clots are best detected on PMMR; the clot shows low T2 signal relative to the high T2 signal of the serum. A postmortem clot can often be distinguished from a central pulmonary embolism that shows a more homogeneous high T1 signal (Fig 6A–6J). Other distinctive features of postmortem clots are that they are seen in the dependent areas of the vessel, usually fill only part of the lumen and do not expand the lumen. With pulmonary embolism the thrombus follows the blood stream until it reaches a point where the lumen becomes too narrow or the vessel branches. The shape of a postmortem clot is often more irregular than a thrombus (Fig 6C and 6D). [60] If clinically relevant, a CT-guided biopsy may help differentiate between postmortem clotting and pulmonary embolism.

In this study we investigated in-hospital deceased adult deaths. The mean PTI was relatively short and bodies were stored in a protected environment after death, PTI seem to have an impact on the occurrence and extent of specific changes.

Autolysis occurs early after death. It leads to significant changes that can be noted at microscopic examination of tissues obtained at biopsy, in particular of the pancreas and adrenal glands. [12, 61] However, in our cohort imaging features of these organs seem less affected by autolysis. Imaging features related to decomposition were seen more frequently. There was tendency to more extensive livores of the lungs with longer PTI and the livores can become so extensive as to completely consolidate the lung parenchyma. In such cases, accurate diagnosis of underlying parenchymal disease can be challenging. In such cases we highly recommend to biopsy both normal and suspected parts of parenchyma to reliably differ postmortem changes from infection (Fig 3A–3I), hemorrhage, or tumor. [13, 14, 62, 63]

The distribution of putrefactive gas also differs with a different PTI, first occurring in the heart cavities and large vessels and with longer intervals in the smaller vessels, organ parenchyma and soft tissues. Putrefactive gas must be differentiated from pathological air collections, such as soft tissue emphysema, free air or gas in the intestinal wall. Putrefactive gas usually has an intestinal origin and travels through the mucosa to the portal veins in the early stage. It may mimic air embolism, however the latter will show a more equal distribution throughout the vascular system. [14] Intestinal bacteria continue to produce gas after death causing bowel distension. The amount of intestinal air significantly increases with longer PTI. This may look similar to a bowel obstruction or paralysis, and should be carefully evaluated. With longer PTI, putrefaction can also lead to formation of subcutaneous air.

Our study had several limitations. We composed a scoring list of postmortem imaging features that may not be complete and some features may be missing. We did not measure body temperature during scanning. Ideally, body temperature should be monitored to allow adaption of MR scan parameters to temperature variations to achieve optimal tissue contrast. However all bodies were stored at the morgue at a constant temperature of 5 degrees Celsius prior to scanning and the transit time from the morgue to the MR scanner was equal for all cases.

We optimized MR sequences for scanning of cold corpses. Furthermore the scan time was maintained approximately the same for all scanning sessions while the temperature in the scanning room was kept constant.

## Conclusion

There is a wide variety of imaging features of postmortem change in in-hospital deaths. These imaging features vary among clinical conditions, increase with longer PTI and must be distinguished from pathologic changes.

## Supporting information

S1 File Dataset PM changes. (SAV)

## References

[1] Burton JL, Underwood J. Clinical, educational, and epidemiological value of autopsy. Lancet. 2007;369(9571):1471–80. doi: 10.1016/S0140-6736(07)60376-6 . 17467518 10.1016/S0140-6736(07)60376-6

[2] Gaensbacher S, Waldhoer T, Berzlanovich A. The slow death of autopsies: a retrospective analysis of the autopsy prevalence rate in Austria from 1990 to 2009. Eur J Epidemiol. 2012;27(7):577–80. doi: 10.1007/s10654-012-9709-3 . 22736188 10.1007/s10654-012-9709-3

[3] Turnbull A, Martin J, Osborn M. The death of autopsy? Lancet. 2015;386(10009):2141 doi: 10.1016/S0140-6736(15)01049-1 . 26638959 10.1016/S0140-6736(15)01049-1

[4] Shojania KG, Burton EC, McDonald KM, Goldman L. Changes in rates of autopsy-detected diagnostic errors over time: a systematic review. JAMA. 2003;289(21):2849–56. doi: 10.1001/jama.289.21.2849 . 12783916 10.1001/jama.289.21.2849

[5] Roberts IS, Benamore RE, Benbow EW, Lee SH, Harris JN, Jackson A, et al Post-mortem imaging as an alternative to autopsy in the diagnosis of adult deaths: a validation study. Lancet. 2012;379(9811):136–42. doi: 10.1016/S0140-6736(11)61483-9 ; PubMed Central PMCID: PMCPMC3262166. 22112684 10.1016/S0140-6736(11)61483-9 PMC3262166

[6] Burke M, O'Donnell C. Postmortem Computed Tomography Findings in Ruptured Splenic Artery Aneurysm: Value of the Clinical "Sentinel Clot" Sign in Identification of Bleeding Location. Am J Forensic Med Pathol. 2015;36(3):224–6. doi: 10.1097/PAF.0000000000000180 . 26164764 10.1097/PAF.0000000000000180

[7] Thali MJ, Jackowski C, Oesterhelweg L, Ross SG, Dirnhofer R. VIRTOPSY—the Swiss virtual autopsy approach. Leg Med (Tokyo). 2007;9(2):100–4. doi: 10.1016/j.legalmed.2006.11.011 . 17275386 10.1016/j.legalmed.2006.11.011

[8] Thali MJ, Braun M, Buck U, Aghayev E, Jackowski C, Vock P, et al VIRTOPSY—scientific documentation, reconstruction and animation in forensic: individual and real 3D data based geo-metric approach including optical body/object surface and radiological CT/MRI scanning. J Forensic Sci. 2005;50(2):428–42. . 15813556

[9] Weustink AC, Hunink MG, van Dijke CF, Renken NS, Krestin GP, Oosterhuis JW. Minimally invasive autopsy: an alternative to conventional autopsy? Radiology. 2009;250(3):897–904. doi: 10.1148/radiol.2503080421 . 19244053 10.1148/radiol.2503080421

[10] Blokker BM, Wagensveld IM, Weustink AC, Oosterhuis JW, Hunink MG. Non-invasive or minimally invasive autopsy compared to conventional autopsy of suspected natural deaths in adults: a systematic review. Eur Radiol. 2015 doi: 10.1007/s00330-015-3908-8 . 26210206 10.1007/s00330-015-3908-8 PMC4778156

[11] Eriksson A, Gustafsson T, Hoistad M, Hultcrantz M, Jacobson S, Mejare I, et al Diagnostic accuracy of postmortem imaging vs autopsy-A systematic review. Eur J Radiol. 2016 doi: 10.1016/j.ejrad.2016.08.003 . 28089245 10.1016/j.ejrad.2016.08.003

[12] Charlier P, Carlier R, Roffi F, Ezra J, Chaillot PF, Duchat F, et al Postmortem abdominal CT: assessing normal cadaveric modifications and pathological processes. Eur J Radiol. 2012;81(4):639–47. doi: 10.1016/j.ejrad.2011.01.054 . 21296512 10.1016/j.ejrad.2011.01.054

[13] Klein WM, Bosboom DG, Koopmanschap DH, Nievelstein RA, Nikkels PG, van Rijn RR. Normal pediatric postmortem CT appearances. Pediatr Radiol. 2015;45(4):517–26. doi: 10.1007/s00247-014-3258-8 . 25828355 10.1007/s00247-014-3258-8

[14] Flach PM, Thali MJ, Germerott T. Times have changed! Forensic radiology—a new challenge for radiology and forensic pathology. AJR Am J Roentgenol. 2014;202(4):W325–34. doi: 10.2214/AJR.12.10283 . 24660730 10.2214/AJR.12.10283

[15] Kobayashi M, Takatori T, Iwadate K, Nakajima M. Reconsideration of the sequence of rigor mortis through postmortem changes in adenosine nucleotides and lactic acid in different rat muscles. Forensic Sci Int. 1996;82(3):243–53. . 8948133 10.1016/s0379-0738(96)02005-1

[16] Krompecher T. Experimental evaluation of rigor mortis. V. Effect of various temperatures on the evolution of rigor mortis. Forensic Sci Int. 1981;17(1):19–26. . 7216078 10.1016/0379-0738(81)90184-5

[17] Ampanozi G, Hatch GM, Flach PM, Thali MJ, Ruder TD. Postmortem magnetic resonance imaging: Reproducing typical autopsy heart measurements. Leg Med (Tokyo). 2015;17(6):493–8. doi: 10.1016/j.legalmed.2015.10.008 . 26593996 10.1016/j.legalmed.2015.10.008

[18] Shiotani S, Kohno M, Ohashi N, Yamazaki K, Nakayama H, Watanabe K, et al Dilatation of the heart on postmortem computed tomography (PMCT): comparison with live CT. Radiat Med. 2003;21(1):29–35. . 12801141

[19] Zech WD, Schwendener N, Persson A, Warntjes MJ, Jackowski C. Temperature dependence of postmortem MR quantification for soft tissue discrimination. Eur Radiol. 2015;25(8):2381–9. doi: 10.1007/s00330-015-3588-4 . 25636417 10.1007/s00330-015-3588-4

[20] Roberts IS, Benbow EW, Bisset R, Jenkins JP, Lee SH, Reid H, et al Accuracy of magnetic resonance imaging in determining cause of sudden death in adults: comparison with conventional autopsy. Histopathology. 2003;42(5):424–30. . 12713618 10.1046/j.1365-2559.2003.01614.x

[21] Dirnhofer R, Jackowski C, Vock P, Potter K, Thali MJ. VIRTOPSY: minimally invasive, imaging-guided virtual autopsy. Radiographics. 2006;26(5):1305–33. doi: 10.1148/rg.265065001 . 16973767 10.1148/rg.265065001

[22] Yamazaki K, Shiotani S, Ohashi N, Doi M, Honda K. Hepatic portal venous gas and hyper-dense aortic wall as postmortem computed tomography finding. Leg Med (Tokyo). 2003;5 Suppl 1:S338–41. . 12935627 10.1016/s1344-6223(02)00166-9

[23] Christe A, Flach P, Ross S, Spendlove D, Bolliger S, Vock P, et al Clinical radiology and postmortem imaging (Virtopsy) are not the same: Specific and unspecific postmortem signs. Leg Med (Tokyo). 2010;12(5):215–22. doi: 10.1016/j.legalmed.2010.05.005 . 20630787 10.1016/j.legalmed.2010.05.005

[24] Jackowski C, Hofmann K, Schwendener N, Schweitzer W, Keller-Sutter M. Coronary thrombus and peracute myocardial infarction visualized by unenhanced postmortem MRI prior to autopsy. Forensic Sci Int. 2012;214(1–3):e16–9. doi: 10.1016/j.forsciint.2011.07.010 . 21802227 10.1016/j.forsciint.2011.07.010

[25] Scholing M, Saltzherr TP, Fung Kon Jin PH, Ponsen KJ, Reitsma JB, Lameris JS, et al The value of postmortem computed tomography as an alternative for autopsy in trauma victims: a systematic review. Eur Radiol. 2009;19(10):2333–41. doi: 10.1007/s00330-009-1440-4 ; PubMed Central PMCID: PMCPMC2758189. 19458952 10.1007/s00330-009-1440-4 PMC2758189

[26] Egger C, Vaucher P, Doenz F, Palmiere C, Mangin P, Grabherr S. Development and validation of a postmortem radiological alteration index: the RA-Index. Int J Legal Med. 2012;126(4):559–66. doi: 10.1007/s00414-012-0686-6 . 22402872 10.1007/s00414-012-0686-6

[27] Egger C, Bize P, Vaucher P, Mosimann P, Schneider B, Dominguez A, et al Distribution of artifactual gas on post-mortem multidetector computed tomography (MDCT). Int J Legal Med. 2012;126(1):3–12. doi: 10.1007/s00414-010-0542-5 . 21207230 10.1007/s00414-010-0542-5

[28] Hyodoh H, Sato T, Onodera M, Washio H, Hasegawa T, Hatakenaka M. Vascular measurement changes observed using postmortem computed tomography. Jpn J Radiol. 2012;30(10):840–5. doi: 10.1007/s11604-012-0134-z . 23054882 10.1007/s11604-012-0134-z

[29] Jackowski C, Grabherr S, Schwendener N. Pulmonary thrombembolism as cause of death on unenhanced postmortem 3T MRI. Eur Radiol. 2013;23(5):1266–70. doi: 10.1007/s00330-012-2728-3 . 23242001 10.1007/s00330-012-2728-3

[30] Jackowski C, Thali M, Aghayev E, Yen K, Sonnenschein M, Zwygart K, et al Postmortem imaging of blood and its characteristics using MSCT and MRI. Int J Legal Med. 2006;120(4):233–40. doi: 10.1007/s00414-005-0023-4 . 16328426 10.1007/s00414-005-0023-4

[31] Kobayashi T, Shiotani S, Kaga K, Saito H, Saotome K, Miyamoto K, et al Characteristic signal intensity changes on postmortem magnetic resonance imaging of the brain. Jpn J Radiol. 2010;28(1):8–14. doi: 10.1007/s11604-009-0373-9 . 20112087 10.1007/s11604-009-0373-9

[32] Levy AD, Harcke HT, Getz JM, Mallak CT, Caruso JL, Pearse L, et al Virtual autopsy: two- and three-dimensional multidetector CT findings in drowning with autopsy comparison. Radiology. 2007;243(3):862–8. doi: 10.1148/radiol.2433061009 . 17517939 10.1148/radiol.2433061009

[33] Takahashi N, Satou C, Higuchi T, Shiotani M, Maeda H, Hirose Y. Quantitative analysis of intracranial hypostasis: comparison of early postmortem and antemortem CT findings. AJR Am J Roentgenol. 2010;195(6):W388–93. doi: 10.2214/AJR.10.4442 . 21098169 10.2214/AJR.10.4442

[34] Scheurer E, Lovblad KO, Kreis R, Maier SE, Boesch C, Dirnhofer R, et al Forensic application of postmortem diffusion-weighted and diffusion tensor MR imaging of the human brain in situ. AJNR Am J Neuroradiol. 2011;32(8):1518–24. doi: 10.3174/ajnr.A2508 . 21659482 10.3174/ajnr.A2508 PMC7964364

[35] Smith AB, Lattin GE Jr., Berran P., Harcke HT. Common and expected postmortem CT observations involving the brain: mimics of antemortem pathology. AJNR Am J Neuroradiol. 2012;33(7):1387–91. doi: 10.3174/ajnr.A2966 . 22492568 10.3174/ajnr.A2966 PMC7965507

[36] Jackowski C, Schweitzer W, Thali M, Yen K, Aghayev E, Sonnenschein M, et al Virtopsy: postmortem imaging of the human heart in situ using MSCT and MRI. Forensic Sci Int. 2005;149(1):11–23. doi: 10.1016/j.forsciint.2004.05.019 . 15734105 10.1016/j.forsciint.2004.05.019

[37] Jackowski C, Christe A, Sonnenschein M, Aghayev E, Thali MJ. Postmortem unenhanced magnetic resonance imaging of myocardial infarction in correlation to histological infarction age characterization. Eur Heart J. 2006;27(20):2459–67. doi: 10.1093/eurheartj/ehl255 . 16973689 10.1093/eurheartj/ehl255

[38] Thali MJ, Yen K, Schweitzer W, Vock P, Ozdoba C, Dirnhofer R. Into the decomposed body-forensic digital autopsy using multislice-computed tomography. Forensic Sci Int. 2003;134(2–3):109–14. . 12850403 10.1016/s0379-0738(03)00137-3

[39] Eggen MD, Swingen CM, Iaizzo PA. Ex vivo diffusion tensor MRI of human hearts: relative effects of specimen decomposition. Magn Reson Med. 2012;67(6):1703–9. doi: 10.1002/mrm.23194 . 22114027 10.1002/mrm.23194

[40] Jackowski C, Schwendener N, Grabherr S, Persson A. Post-mortem cardiac 3-T magnetic resonance imaging: visualization of sudden cardiac death? J Am Coll Cardiol. 2013;62(7):617–29. doi: 10.1016/j.jacc.2013.01.089 . 23563129 10.1016/j.jacc.2013.01.089

[41] Crooijmans HJ, Ruder TD, Zech WD, Somaini S, Scheffler K, Thali MJ, et al Cardiovascular magnetization transfer ratio imaging compared with histology: a postmortem study. J Magn Reson Imaging. 2014;40(4):915–9. doi: 10.1002/jmri.24460 . 24227690 10.1002/jmri.24460

[42] Shiotani S, Kohno M, Ohashi N, Yamazaki K, Nakayama H, Watanabe K, et al Non-traumatic postmortem computed tomographic (PMCT) findings of the lung. Forensic Sci Int. 2004;139(1):39–48. . 14687772 10.1016/j.forsciint.2003.09.016

[43] O'Donnell C, Woodford N. Post-mortem radiology—a new sub-speciality? Clin Radiol. 2008;63(11):1189–94. doi: 10.1016/j.crad.2008.05.008 . 18929036 10.1016/j.crad.2008.05.008

[44] Burke M, Parsons S, Bassed R. Management of medicolegal natural deaths from hemopericardium or hemothorax using postmortem CT scanning. Forensic Sci Med Pathol. 2012;8(4):367–72. doi: 10.1007/s12024-012-9347-9 . 22644883 10.1007/s12024-012-9347-9

[45] Ross SG, Thali MJ, Bolliger S, Germerott T, Ruder TD, Flach PM. Sudden death after chest pain: feasibility of virtual autopsy with postmortem CT angiography and biopsy. Radiology. 2012;264(1):250–9. doi: 10.1148/radiol.12092415 . 22570504 10.1148/radiol.12092415

[46] Bruguier C, Mosimann PJ, Vaucher P, Uske A, Doenz F, Jackowski C, et al Multi-phase postmortem CT angiography: recognizing technique-related artefacts and pitfalls. Int J Legal Med. 2013;127(3):639–52. doi: 10.1007/s00414-013-0840-9 . 23515679 10.1007/s00414-013-0840-9

[47] Ruder TD, Thali MJ, Hatch GM. Essentials of forensic post-mortem MR imaging in adults. Br J Radiol. 2014;87(1036):20130567 doi: 10.1259/bjr.20130567 ; PubMed Central PMCID: PMCPMC4067017. 24191122 10.1259/bjr.20130567 PMC4067017

[48] Jackowski C, Sonnenschein M, Thali MJ, Aghayev E, Yen K, Dirnhofer R, et al Intrahepatic gas at postmortem computed tomography: forensic experience as a potential guide for in vivo trauma imaging. J Trauma. 2007;62(4):979–88. doi: 10.1097/01.ta.0000198733.22654.de . 17426557 10.1097/01.ta.0000198733.22654.de

[49] Germerott T, Preiss US, Ebert LC, Ruder TD, Ross S, Flach PM, et al A new approach in virtopsy: Postmortem ventilation in multislice computed tomography. Leg Med (Tokyo). 2010;12(6):276–9. doi: 10.1016/j.legalmed.2010.07.001 . 20729123 10.1016/j.legalmed.2010.07.001

[50] Michiue T, Sakurai T, Ishikawa T, Oritani S, Maeda H. Quantitative analysis of pulmonary pathophysiology using postmortem computed tomography with regard to the cause of death. Forensic Sci Int. 2012;220(1–3):232–8. doi: 10.1016/j.forsciint.2012.03.007 . 22503888 10.1016/j.forsciint.2012.03.007

[51] Jackowski C, Thali MJ, Buck U, Aghayev E, Sonnenschein M, Yen K, et al Noninvasive estimation of organ weights by postmortem magnetic resonance imaging and multislice computed tomography. Invest Radiol. 2006;41(7):572–8. doi: 10.1097/01.rli.0000221323.38443.8d . 16772850 10.1097/01.rli.0000221323.38443.8d

[52] Arthurs OJ, Barber JL, Taylor AM, Sebire NJ. Normal perinatal and paediatric postmortem magnetic resonance imaging appearances. Pediatr Radiol. 2015;45(4):527–35. Epub 2015/04/02. doi: 10.1007/s00247-014-3166-y ; PubMed Central PMCID: PMC4381098. 25828356 10.1007/s00247-014-3166-y PMC4381098

[53] Gebhart FT, Brogdon BG, Zech WD, Thali MJ, Germerott T. Gas at postmortem computed tomography—an evaluation of 73 non-putrefied trauma and non-trauma cases. Forensic Sci Int. 2012;222(1–3):162–9. doi: 10.1016/j.forsciint.2012.05.020 . 22721934 10.1016/j.forsciint.2012.05.020

[54] Fischer F, Grimm J, Kirchhoff C, Reiser MF, Graw M, Kirchhoff S. Postmortem 24-h interval computed tomography findings on intrahepatic gas development and changes of liver parenchyma radiopacity. Forensic Sci Int. 2012;214(1–3):118–23. doi: 10.1016/j.forsciint.2011.07.033 . 21862251 10.1016/j.forsciint.2011.07.033

[55] Okuda T, Shiotani S, Kobayashi T, Kohno M, Hayakawa H, Kikuchi K, et al Immediate non-traumatic postmortem computed tomographic demonstration of myocardial intravascular gas of the left ventricle: effects from cardiopulmonary resuscitation. Springerplus. 2013;2(1):86 doi: 10.1186/2193-1801-2-86 ; PubMed Central PMCID: PMCPMC3599202. 23519017 10.1186/2193-1801-2-86 PMC3599202

[56] Shiotani S, Ueno Y, Atake S, Kohno M, Suzuki M, Kikuchi K, et al Nontraumatic postmortem computed tomographic demonstration of cerebral gas embolism following cardiopulmonary resuscitation. Jpn J Radiol. 2010;28(1):1–7. doi: 10.1007/s11604-009-0372-x . 20112086 10.1007/s11604-009-0372-x

[57] Takahashi N, Satou C, Higuchi T, Shiotani M, Maeda H, Hirose Y. Quantitative analysis of brain edema and swelling on early postmortem computed tomography: comparison with antemortem computed tomography. Jpn J Radiol. 2010;28(5):349–54. doi: 10.1007/s11604-010-0430-4 . 20585922 10.1007/s11604-010-0430-4

[58] Kanazawa A, Hyodoh H, Watanabe S, Fukuda M, Baba M, Okazaki S, et al New pitfalls of high-density postmortem computed tomography. Leg Med (Tokyo). 2014;16(5):297–9. doi: 10.1016/j.legalmed.2014.05.004 . 24916862 10.1016/j.legalmed.2014.05.004

[59] Takahashi N, Higuchi T, Hirose Y, Yamanouchi H, Takatsuka H, Funayama K. Changes in aortic shape and diameters after death: comparison of early postmortem computed tomography with antemortem computed tomography. Forensic Sci Int. 2013;225(1–3):27–31. doi: 10.1016/j.forsciint.2012.04.037 . 22656269 10.1016/j.forsciint.2012.04.037

[60] Schwendener N, Mund M, Jackowski C. Type II DeBakey dissection with complete aortic rupture visualized by unenhanced postmortem imaging. Forensic Sci Int. 2013;225(1–3):67–70. doi: 10.1016/j.forsciint.2012.09.002 . 23021107 10.1016/j.forsciint.2012.09.002

[61] Levy AD, Harcke HT, Mallak CT. Postmortem imaging: MDCT features of postmortem change and decomposition. Am J Forensic Med Pathol. 2010;31(1):12–7. doi: 10.1097/PAF.0b013e3181c65e1a . 20010292 10.1097/PAF.0b013e3181c65e1a

[62] Arthurs OJ, Guy A, Kiho L, Sebire NJ. Ventilated postmortem computed tomography in children: feasibility and initial experience. Int J Legal Med. 2015;129(5):1113–20. doi: 10.1007/s00414-015-1189-z . 25904077 10.1007/s00414-015-1189-z

[63] Shiotani S, Kobayashi T, Hayakawa H, Kikuchi K, Kohno M. Postmortem pulmonary edema: a comparison between immediate and delayed postmortem computed tomography. Leg Med (Tokyo). 2011;13(3):151–5. doi: 10.1016/j.legalmed.2010.12.008 . 21315646 10.1016/j.legalmed.2010.12.008

